# Neural Correlates of Anosognosia in Alzheimer's Disease and Mild Cognitive Impairment: A Multi-Method Assessment

**DOI:** 10.3389/fnbeh.2018.00100

**Published:** 2018-05-17

**Authors:** Manuela Tondelli, Anna M. Barbarulo, Giulia Vinceti, Chiara Vincenzi, Annalisa Chiari, Paolo F. Nichelli, Giovanna Zamboni

**Affiliations:** ^1^Dipartimento di Scienze Biomediche, Metaboliche e Neuroscienze, Università di Modena e Reggio Emilia, Modena, Italy; ^2^Center for Neurosciences and Neurotechnology, Università di Modena e Reggio Emilia, Modena, Italy; ^3^Dipartimento di Cure Primarie, Azienda Unità Sanitaria Locale di Modena, Modena, Italy; ^4^Neurologia, Azienda Ospedaliero Universitaria Policlinico di Modena, Modena, Italy; ^5^Seconda Divisione, Neurologia, Centro per la Sclerosi Multipla, Università Campana Luigi Vanvitelli, Naples, Italy; ^6^Nuffield Department of Clinical Neurosciences, University of Oxford, Oxford, United Kingdom

**Keywords:** anosognosia, unawareness of disease, Mild Cognitive Impairment, Alzheimer's disease, dementia

## Abstract

Patients with Alzheimer's Disease (AD) and Mild Cognitive Impairment (MCI) may present anosognosia for their cognitive deficits. Three different methods have been usually used to measure anosognosia in patients with AD and MCI, but no studies have established if they share similar neuroanatomical correlates. The purpose of this study was to investigate if anosognosia scores obtained with the three most commonly used methods to assess anosognosia relate to focal atrophy in AD and MCI patients, in order to improve understanding of the neural basis of anosognosia in dementia. Anosognosia was evaluated in 27 patients (15 MCI and 12 AD) through clinical rating (Clinical Insight Rating Scale, CIRS), patient-informant discrepancy (Anosognosia Questionnaire Dementia, AQ-D), and performance discrepancy on different cognitive domains (self-appraisal discrepancies, SADs). Voxel-based morphometry correlational analyses were performed on magnetic resonance imaging (MRI) data with each anosognosia score. Increasing anosognosia on any anosognosia measurement (CIRS, AQ-D, SADs) was associated with increasing gray matter atrophy in the medial temporal lobe including the right hippocampus. Our results support a unitary mechanism of anosognosia in AD and MCI, in which medial temporal lobes play a key role, irrespectively of the assessment method used. This is in accordance with models suggesting that anosognosia in AD is primarily caused by a decline in mnemonic processes.

## Introduction

Awareness of our own performance is a critical component of normal cognition that gives us the ability to recognize our limits and plan our behavior accordingly. But patients with Alzheimer's Disease (AD) and its pre-dementia counterpart indicated as Mild Cognitive Impairment (MCI) are frequently unaware of their cognitive and behavioral deficits (Morris and Mograbi, 2013; Sunderaraman and Cosentino, 2017). The inability to recognize cognitive, behavioral, or functional impairment occurring as a consequence of a dementing illness is indicated as *anosognosia, loss of insight*, or *unawareness of disease* (Babinski, 1914). Anosognosia in patients with MCI and AD has significant clinical implications for their care, in that reduces compliance to treatment, threatens patient's safety, and increases caregiver burden (Starkstein et al., 2007). Many previous studies exploring aspects of anosognosia in people with MCI and AD have led to heterogeneous and sometimes inconsistent findings concerning the association of anosognosia with neuropsychological and psychiatric characteristics (Kaszniak and Edmons, 2010; Starkstein et al., 2010; De Carolis et al., 2015), severity of disease (Sunderaraman and Cosentino, 2017), and underlying neuroanatomical correlates (Zamboni and Wilcock, 2011; Cosentino et al., 2015). The cause of this variability may in part reflect different approaches used to study insight, that can be broadly classified in clinical approaches adopting the clinical concept of anosognosia or approaches from cognitive psychology adopting the concept of metacognition (Sunderaraman and Cosentino, 2017). These different approaches have in turn used different methods to measure anosognosia (or metacognition) (Clare et al., 2011). A first method usually indicated as “clinician rating” is based on the judgment of the clinician who rates the patient's level of awareness along an ordinal scale, following structured or unstructured interviews with the patients and the caregiver. The second method usually indicated as “patient-informant discrepancy” is based on the calculation of discrepancy scores between parallel questionnaire for the patient and for their caregiver, in which they both describe the patient's potential symptoms (Hannesdottir and Morris, 2007). These two approaches are commonly used in clinical settings and measure anosognosia in patients with dementia in an *offline* way (Sunderaraman and Cosentino, 2017). The third method usually indicated as “performance discrepancy” is based on the comparison between the patient's actual performance on a certain neuropsychological test and their estimation of how well they think they performed immediately prior to or following the execution of the specific test. Variations of this method have been traditionally used to assess meta-cognitive abilities in healthy subjects in cognitive psychology studies, but have recently been increasingly used to measure *online* and immediate insight in patients with dementia (Martyr et al., 2014).

However, only a few studies have directly compared the three methods in the same sample of patients (Hannesdottir and Morris, 2007; Leicht et al., 2010; Clare et al., 2011). Hannesdottir and Morris used clinician rating, patient-informant discrepancy, and performance discrepancy to assess anosognosia in 92 AD patients. They found that only clinician rating and patient-informant discrepancy were reciprocally correlated, whereas performance discrepancy was related to measures of memory and executive functioning (Hannesdottir and Morris, 2007). The same authors had previously suggested a neuropsychological model of anosognosia that distinguishes between primary anosognosia, which directly affects a long-term system specific for self-awareness, and secondary anosognosia, which instead is secondary to memory or executive dysfunction that affects the immediate ability to judge cognitive performance (Agnew and Morris, 1998; Morris and Hannesdottir, 2004). They concluded that clinician rating and patient-informant discrepancy inform about longer term awareness (i.e., primary anosognosia), whereas performance discrepancy reflects the impairment of the online ability to judge cognitive performance (i.e., secondary anosognosia) (Hannesdottir and Morris, 2007). Another study conducted in 32 patients with AD gave similar results, suggesting that different methods of measurement may capture different facets of anosognosia (Leicht et al., 2010).

Imaging studies looking at the neuroanatomical correlates of anosognosia have typically adopted only one among the three above mentioned methods and correlated it with measures of brain variability, such as metabolism or gray matter atrophy. However, in a review on the neuroanatomical correlates of anosognosia it was observed that studies using performance discrepancy showed associations between anosognosia and atrophy or hypo-metabolism mainly in frontal regions, whereas studies using patient-informant discrepancy more frequently showed involvement of temporo-parietal regions, although the results may have been biased by a tendency to only focus on frontal regions in some of the older studies (Zamboni and Wilcock, 2011). To our knowledge, no imaging studies have simultaneously studied the neuroanatomical correlates of the three different methods most commonly used to assess anosognosia.

Here we investigated the brain correlates of anosognosia assessed with clinical rating, patient-informant discrepancy, and performance discrepancy in a sample of AD and MCI patients. We reasoned that if different measures of anosognosia would have different neuroanatomical correlates it would imply that they capture different aspects and possibly different mechanisms of anosognosia. Alternatively, if different measures of anosognosia would show overlapping neuroanatomical correlates, it would support that anosognosia in MCI and AD can be studied either through the cognitive construct of meta-cognition or the clinical construct of anosognosia. More precisely, we hypothesized that such unitary model would rely on medial temporal structures in line with the most recent revision of the theoretical model of anosognosia, which suggests that anosognosia in AD is mainly caused by a decline in specific mnemonic processes ultimately leading to the loss of personal knowledge (Morris and Mograbi, 2013).

## Materials and methods

### Participants

Twenty-seven elderly participants took part in the study. Fifteen had a diagnosis of amnestic MCI (a-MCI) and 12 had a diagnosis of AD. Exclusion criteria were severe head trauma or prior neurological disorders, major psychiatric disorder, possible vascular or mixed origin of the cognitive dysfunction, and evidence of cerebral lesions on MRI scan, including evidence of small vessel disease (infarcts, lacunes, micro-bleeds, or white matter hyper-intensities). Participant were recruited from the Memory Clinic of the Neurological Department of the Nuovo Ospedale Civile S. Agostino-Estense (Baggiovara, Modena, Italy). They were asked to participate with an informant who knew them well enough to give information about their activities of daily living and cognitive performance. Clinical diagnoses of amnestic MCI and AD were made according to published criteria (McKhann et al., 1984; Petersen et al., 1999; Winblad et al., 2004). Participants underwent medical and neurological examination. neuropsychological assessment, and MRI scan. Participant's study partners were interviewed to gain additional information about subject's cognitive, functional, and behavioral status. The study was conducted under ethical approval of the Local Ethics Committee (Comitato Etico Provinciale di Modena, code 252.09, approved on 21/04/10) and all subjects gave written informed consent before participating. The degree of cognitive impairment was assessed by the Mini-Mental State Examination (MMSE, Folstein et al., 1975). Standard neuropsychological assessment was performed, including measure of verbal fluency, visuospatial and verbal memory, executive functions, and attention. The Clinical Dementia Rating Scale (CDR, Morris, 1993) was administered to the caregiver to measure dementia severity (AD: rated as 1; a-MCI: rated as 0.5). The distress of caregiver was ruled out through the Caregiver Burden Inventory (CBI, Novak and Guest, 1989).

### Assessment of anosognosia

We used 3 methods to assess anosognosia:

1. Clinician ratings on the *Clinical Insight Rating Scale, CIRS* (Ott et al., 1996; Zanetti et al., 1999). The CIRS define 4 domains of patient's awareness: (a) the reason for the visit; (b) the cognitive deficits; (c) functional deficits; (d) perception of the progression of deficits. Based on separate interview with the patient and the caregiver, the items are rated by the examiner as 0 (full insight), or 1 (partial insight), or 2 (non-insight), and summed to obtain a total score between 0 and 8. Patients with a total score ≥2 were considered anosognosic;
2. Patient-informant discrepancy score evaluated by *Anosognosia Questionnaire Dementia (AQ-D*, Migliorelli et al., 1995). The questionnaire consists of 30 questions divided in 2 sections, the cognitive and the behavioral aspects. The cognitive part assesses cognitive function and performance, in basic and instrumental activities of daily living. The behavioral part assesses changes in interests and mood. The same questions were administered to patients (Form A) and to caregivers (Form B) who were blinded to the patient's answers. The total AQ-D score is given by the difference between Form B and Form A. Higher score indicated a reduced awareness of deficits, meaning that caregivers rated the patients more impaired that did the patients;
3. Performance discrepancy was assessed by asking patients to retrospectively rate their own performance on different neuropsychological tests (Leicht et al., 2010; Rosen et al., 2010) then calculating a *Self-appraisal Discrepancy (SAD)* score for each test. We focused on tests of memory and executive function as these cognitive domains have been those most frequently associated with anosognosia (Lopez et al., 1994; Kashiwa et al., 2005; Hannesdottir and Morris, 2007). More precisely we included Babcock Story Recall (Carlesimo et al., 2002) and Rey Auditory-Verbal Learning Test-Delayed Recall (Carlesimo et al., 1996) for verbal memory, Rey-Osterrieth Complex Figure-Recall (Bertolani et al., 1993) for visuo-spatial memory, the Stroop Test (Caffarra et al., 2002), and the Frontal Assessment Battery (FAB, Appollonio et al., 2005) for executive function. Patients were asked to rate their perception of performance on these tests by assigning themselves a score on a 5-point scale ranging from 0 (very poor) to 4 (very good). Raw scores on each of the cognitive tasks were converted into equivalent score using published norms. The performance score was subtracted from the self-rating score, resulting in a *Self-appraisal Discrepancy (SAD)* score between −4 (underestimation of performance) and +4 (overestimation of performance), with zero representing perfect accuracy.

### Statistical analyses

Analyses of behavioral and neuropsychological data were performed with SPSS version 24.0. Comparisons between MCI and AD groups were performed with Mann-Whitney or independent *t*-test, as appropriate, for continuous variables, and chi-square tests for dichotomous variables, using a level of statistical significance of *p* < 0.006 to control for the number of comparisons (8, including MMSE and all the measures of anosognosia). Correlations among measures of anosognosia and between these and neuropsychological tests were also performed, using a level of statistical significance of *p* < 0.003 to control for the number of correlations (14). Pearson's and Spearman's correlation test were used according to the linearity of the relationship between variables.

### Images acquisition and analysis

Scanning was performed at Nuovo Ospedale Civile S.Agostino, Estense, Baggiovara, Modena using a 3-T Philips Intera MRI scanner equipped with a 12-channel head coil. The images acquired were high-resolution T1-weighted 3D MP-RAGE structural images (repetition time 9,900 ms; echo time 4.6 ms; field of view 256 × 256 mm; voxel dimension 1 mm^3^). Structural data were analyzed with FSL-VBM, a voxel-based morphometry style analysis carried out with FSL tools (http://www.fmrib.ox.ac.uk) (Smith et al., 2004), to detect gray matter (GM) differences. First, structural images were brain-extracted and gray matter segmented before being registered to the MNI 152 standard space using non-linear registration (Andersson et al., 2007). The resulting images were averaged to create a study-specific template, to which the native gray matter segmented images were then non-linearly re-registered. The method also introduces a compensation (or “modulation”) for the contraction/enlargement due to the non-linear component of the transformation: each voxel of each registered gray matter image was divided by the Jacobian of the warp field. The modulated, registered gray matter images were then smoothed with an isotropic Gaussian kernel with a sigma of 3 mm. Finally, voxelwise General Linear Modeling (GLM) was applied using permutation-based non-parametric testing (5,000 permutations). We performed correlation analyses across the whole cohort of subjects to examine the relationship between GM volume and anosognosia assessed by CIRS, AQ-D and SAD with separate independent GLM models. In all these GLM models, a measure of disease severity (MMSE scores) and age were also mean-centered and entered as covariates of no interest to control for their potential effects. One-tailed *t*-tests were performed, assuming that higher AQ-D, CIRS or SAD scores (indicating higher anosognosia) would be associated with decreased gray matter density (or increased focal atrophy). Results from the different correlational analyses were thresholded, binarised and overlapped to obtain a density overlap showing common results across different measures of anosognosia. To definitively rule out an effect of severity of group on the results, we also conducted separate correlation analyses in the AD and MCI groups with the AQ-D.

Results were first explored at the uncorrected voxel-level using a threshold of *p* < 0.001, then also after applying correction for multiple comparisons at the *p* < 0.05 level (Threshold-Free Cluster Enhancement, TFCE).

## Results

### Behavioral results

There were no significant differences in age, years of education, reported duration of cognitive complain, and gender between the AD and MCI groups (Table 1). As expected, global cognitive decline measured with MMSE was higher in the AD relative to the MCI group (*p* = 0.004). Patients with AD had higher scores in AQ-D and CIRS relative to MCI, but the difference did not reach a statistical significance after correction for multiple comparisons. All AD patients had mean positive discrepancy score in SAD, indicating that they overestimated their performance.

**Table 1:** Demographic and neuropsychological characteristics of participants.

	**Whole group (*n* = 27)**	**MCI (*n* = 15)**	**AD (*n* = 12)**	**Groups comparison**
Gender F:M	15:12	9:6	6:6	*p* = 0.6
Age (years)	75.89 *(6.22)*	76.73 (5.3)	74.77 (7.2)	*p* = 0.46
Education (years)	5.9 *(3.4)*	6.6 (3.9)	5.1 (2.6)	*p* = 0.24
Reported duration of cognitive complain (years)	3.55 *(1.9)*	3.86 (2.41)	3.16 (1.19)	*p* = 0.71
MMSE	25.59 (3.2)	27.3 (0.4)	23.4 (1.0)	***p*** = **0.004**
AQ-D	2.1 (12.3)	−3.0 (9.3)	7.7 (13.1)	*p* = 0.03
CIRS	1.4 (1.7)	0.8 (1.18)	2.2 (2.13)	*p* = 0.04
SAD-Babcock Story Recall	−0.03 (1.8)	−0.60 (2.2)	0.66 (0.77)	*p* = 0.08
SAD-Rey Auditory-Verbal Learning Test-Delayed Recall	0.22 (1.28)	−0.2 (1.9)	0.8 (1.02)	*p* = 0.02
SAD-Rey-Osterrieth Complex Figure-Recall	−0.26 (1.63)	−0.6 (1.9)	0.16 (1.02)	*p* = 0.21
SAD-Frontal Assessment Battery	0.88 (1.6)	0.6 (1.6)	1.25 (1.48)	*p* = 0.30
SAD-Stroop Test	1.11 (1.69)	−0.2 (1.38)	0.58 (1.97)	*p* = 0.20

*Reported values are means with standard deviation values in parenthesis. MMSE, Mini-Mental-State Examination. AQ-D, Anosognosia Questionnaire Dementia; CIRS, Clinical Insight Rating Scale; SAD, Self-Appraisal Discrepancy; F, Female; M, Male. Mann-Whitney test was used for duration of cognitive complaint and SAD scores (italic). In bold, p < 0.006*.

Correlation analysis between measures of anosognosia showed significant positive correlations between AQ-D and CIRS (Sperman's Rho, *r*_s_ = 0.61, *p* = 0.001, Table 2) and between AQ-D and FAB-SAD (Sperman's Rho, *r*_s_ = 0.59, *p* = 0.002, Table 2). There was a negative correlation between SAD-Stroop and MMSE (Sperman's Rho, *r*_s_ = −0.60, *p* = 0.001), whereas all other measures of anosognosia did not correlate with severity of disease. AQ-D negatively correlated with actual performance on the FAB (Sperman's Rho, *r*_s_ = −0.75, *p* < 0.001), suggesting a relation with executive dysfunction. AQ-D and CIRS showed no other significant correlations with neuropsychological tests. Self-appraisal discrepancy (SAD) scores all significantly negatively correlated with actual performances on their respective tests, indicating that the higher the SAD, the poorer the performance in the cognitive test (Table 2).

**Table 2:** Correlations between measures of anosognosia and neuropsychological tests.

AQ-D	1													
CIRS	0.61	1									Spearman Correlation 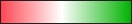 −1.0 0.0 1.0			
SAD-RAVLT Delayed Recall	0.355	0.339	1											
SAD-Babcock Story Recall	0.202	0.352	0.251	1										
SAD-Rey-Osterrieth Complex Figure-Recall	0.419	0.309	0.403	0.227	1									
SAD-FAB	0.588	0.344	0.275	0.191	0.249	1								
SAD-Stroop Test	0.248	0.162	0.425	0.106	0.362	−0.047	1							
Babcock Story Recall	−0.263	−0.364	−0.287	−0.658	−0.282	−0.222	−0.051	1						
RAVLT Delayed Recall	−0.214	−0.395	−0.618	−0.28	−0.344	−0.055	−0.257	0.316	1					
Rey-Osterrieth Complex Figure-Recall	−0.383	−0.387	−0.236	−0.11	−0.513	0.056	−0.338	0.288	0.429	1				
FAB	−0.748	−0.485	−0.438	−0.153	−0.23	−0.815	−0.169	0.322	0.263	0.143	1			
Stroop Time	0.353	0.41	0.559	0.346	0.226	0.226	0.097	−0.357	−0.406	0.103	−0.31	1		
Stroop Errors	0.251	0.186	0.407	0.054	0.317	0.023	0.732	−0.042	−0.201	−0.346	−0.303	0.14	1	
MMSE	−0.423	−0.214	−0.161	−0.211	−0.419	−0.071	−0.596	0.445	0.213	0.53	0.338	−0.055	−0.462	1
	AQD	CIRS	SAD-RAVLT Delayed Recall	SAD-Babcock Story Recall	SAD-Rey-Osterrieth Complex Figure-Recall	SAD-FAB	SAD-Stroop Test	Babcock Story Recall	RAVLT Delayed Recall	Rey-Osterrieth Complex Figure-Recall	FAB	Stroop Time	Stroop Errors	MMSE

*AQ-D, Anosognosia Questionnaire for Dementia; CIRS, Clinical Insight Rating Scale; SAD-RAVLT, Self-Assessment Discrepancy on Rey Auditory-Verbal Learning Test-Delayed Recall; SAD-FAB, Self-Assessment Discrepancy on Frontal Assessment Battery; MMSE, Mini-Mental State Examination. In bold, results significant at p < 0.003*.

### Imaging results

**Figure 1:**
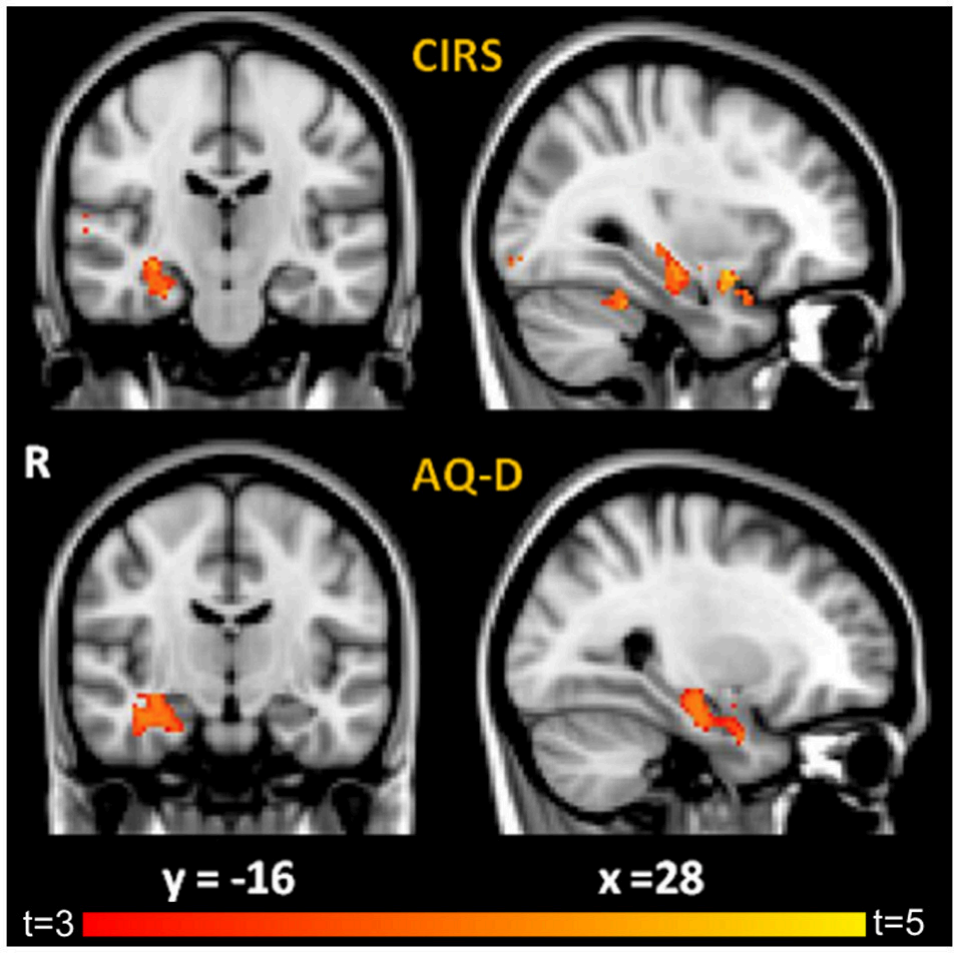
Results of VBM correlation analyses between GM density and: (i) CIRS **(Top)** and (ii) AQ-D **(Bottom)**. Maps of *t*-values are thresholded at *p* < 0.05 corrected for multiple comparisons for AQ-D and at *p* < 0.001 uncorrected for CIRS for display purposes. Images are shown in radiological convention. Coordinates are in MNI.

Correlation analysis between patient-informant discrepancy (AQ-D) and gray matter volume across all subjects showed that higher AQ-D scores (indicating more severe anosognosia) were associated with greater atrophy in the right medial-temporal lobe, in particular the hippocampus (*p* < 0.05, corrected, age and MMSE entered as covariates, Figure 1, Table 3). Correlation analysis between clinical rating (CIRS) and gray matter volume across all subjects gave similar but less significant results, demonstrating that higher CIRS scores (indicating more severe anosognosia) are specifically associated with greater gray matter atrophy in structures of the right medial temporal lobe (*p* < 0.001, uncorrected, age and MMSE entered as covariates).

**Table 3:** Regions of significant negative correlation between GM density and CIRS, AQ-D, and SAD scores.

**Localization**	**Side**	**Cluster size**	**x**	**y**	**z**	***t***	***p***
**AQD**							
Hippocampus	R	595	28	−18	−20	3.94	0.024 corr
Amygdala			32	−2	−22	3.44	
Parahippocampus			36	−14	−24	3.66	
Supramarginal gyrus	R	98	50	−38	34	5.08	0.024 corr
Precentral gyrus	R	78	64	−4	26	4.7	0.030 corr
**SAD-FAB**							
Anterior Cingulate	L	581	−4	−12	44	3.5	0.020 corr
Precentral gyrus			−24	−8	50	3.9	
Postcentral gyrus	R	645	16	−34	72	4.3	0.013 corr
Hippocampus	R	121	30	−22	−18	4.7	0.049 corr
**CIRS**							
Hippocampus	R	116	30	−20	−18	3.4	< 0.001 uncorrected
Amygdala	R	108	32	0	−20	2.9	
Parahippocampus	R	355	24	−28	20	2.7	
Amygdala	L	77	−34	6	−20	2.9	
**SAD-RAVLT**							
Superior frontal gyrus	R	11	26	62	20	3.5	< 0.001 uncorrected
Amygdala	R	15	26	−6	18	3.9	
Hippocampus	R	78	28	−12	−18	4.4	
Parahippocampal gyrus	R	33	28	−30	18	3.4	
Parahipp	L	28	−18	−42	4	4	
Superior temporal gyrus	L	109	−42	−30	4	5.4	
**SAD-Babcock**							
Amygdala	L	298	−24	−8	−14	3.2	< 0.001 uncorrected
Hippocampus	L	430	−22	−16	−22	2.9	
Hippocampus	R	44	26	−16	−18	2.9	
**SAD-Rey Figure Recall**							
Hippocampus	R	114	30	−14	−16	3.7	< 0.001 uncorrected
Lateral occipital cortex. superior divisio	R	322	32	−80	28	4.73	
Precuneus	L	68	−8	−58	32	3.7	
**SAD-Stroop**							
Frontal Orbital Cortex	L	444	−34	20	−26	4.1	< 0.001 uncorrected
Fusiform gyrus	L	665	−38	−64	−18	4.5	
Parahippocampal gyrus	R	94	28	2	−18	3.4	

**Figure 2:**
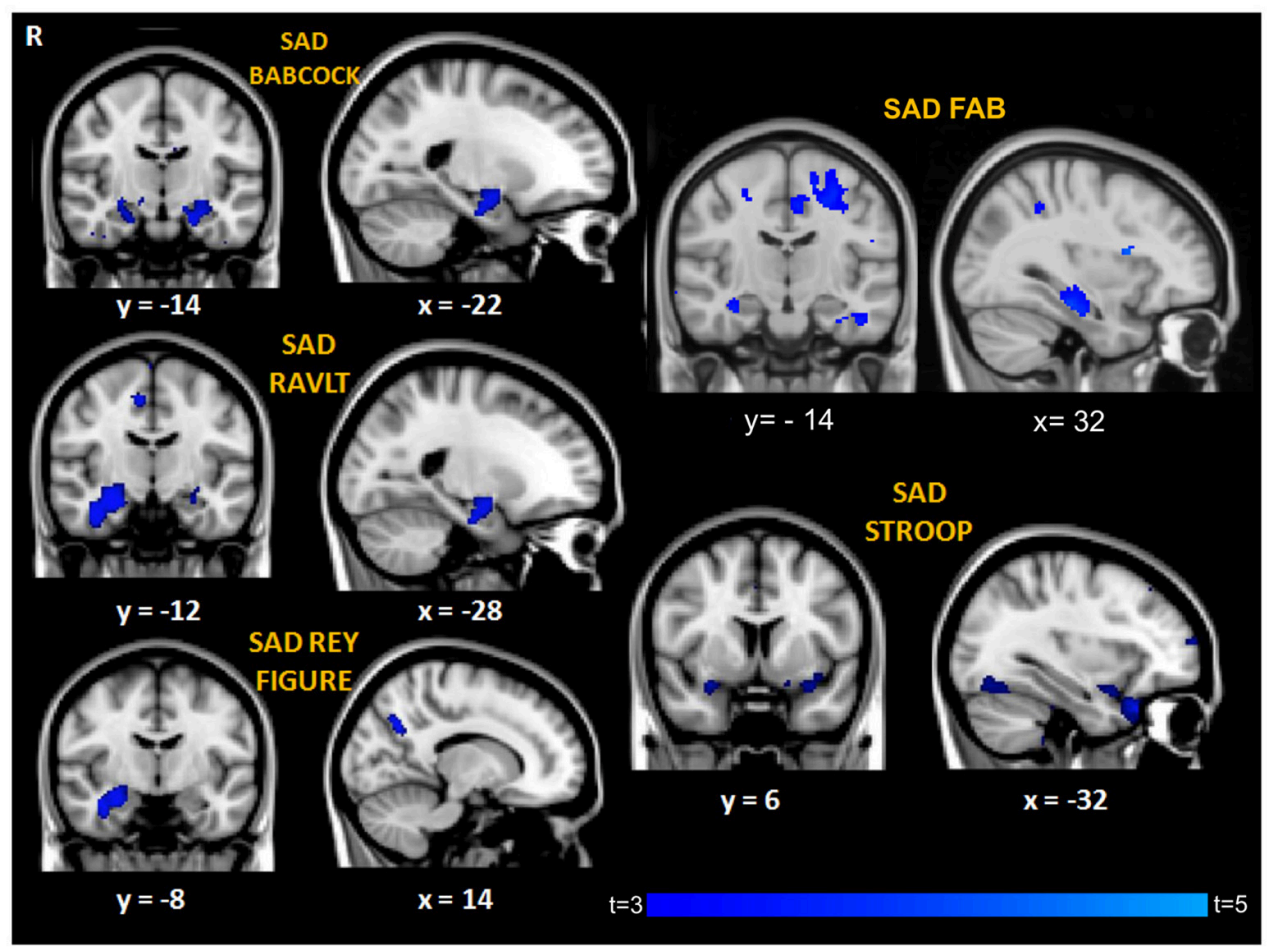
Regions of significant correlation between GM density and SAD for the different cognitive domains. Maps of *t*-values are thresholded at *p* < 0.001 uncorrected, except for SAD-FAB thresholded at *p* < 0.05, corrected for multiple comparison. Images are shown in radiological convention. Coordinates are in MNI.

Correlation analyses between performance discrepancy (SAD scores) and gray matter density showed for all the tests a significant correlation between higher SAD scores (indicating more severe agnosia) and greater atrophy in the right hippocampus (*p* < 0.05 corrected for SAD-FAB, *p* < 0.001, uncorrected for all the other SAD scores; age and MMSE entered as covariates; Figure 2, Table 3). In addition to this significant association with structures of the right medial temporal lobe, higher SAD scores of verbal memory (RAVLT and Babcock Story Recall) were also associated with greater atrophy in the left hippocampus and amygdala bilaterally (*p* < 0.001, uncorrected). Higher SAD score of visuospatial memory (Rey-Osterrieth Complex Figure-Recall) was also associated with greater atrophy in the precuneus and lateral occipital cortex (*p* < 0.001, uncorrected). Higher SAD scores of executive and attentive functions were also associated with greater atrophy in the anterior cingulate gyrus, precentral gyrus, and fronto-orbital cortex (Figure 2, Table 3).

**Figure 3:**
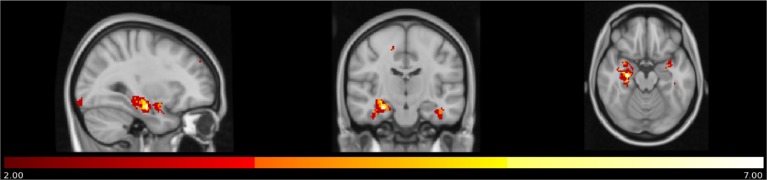
Map of overlap between results (uncorrected *p*) of the correlational analyses on the 7 different measures of anosognosia. In yellow regions in which all the 7 measures of anosognosia correlate with atrophy. In red regions in which at least 2 measures of anosognosia correlate with atrophy.

A density map obtained by overlapping the uncorrected results of the seven different correlational analyses showed that a small region in the right anterior hippocampus (cluster size: 6 voxels, MNI coordinates: 28, −14, −20) resulted from all the different measures of anosognosia and that a larger region extending to the posterior right hippocampus (cluster size: 37 voxels, same center of gravity) resulted from 5 different measures of anosognosia (Figure 3).

**Figure 4:**
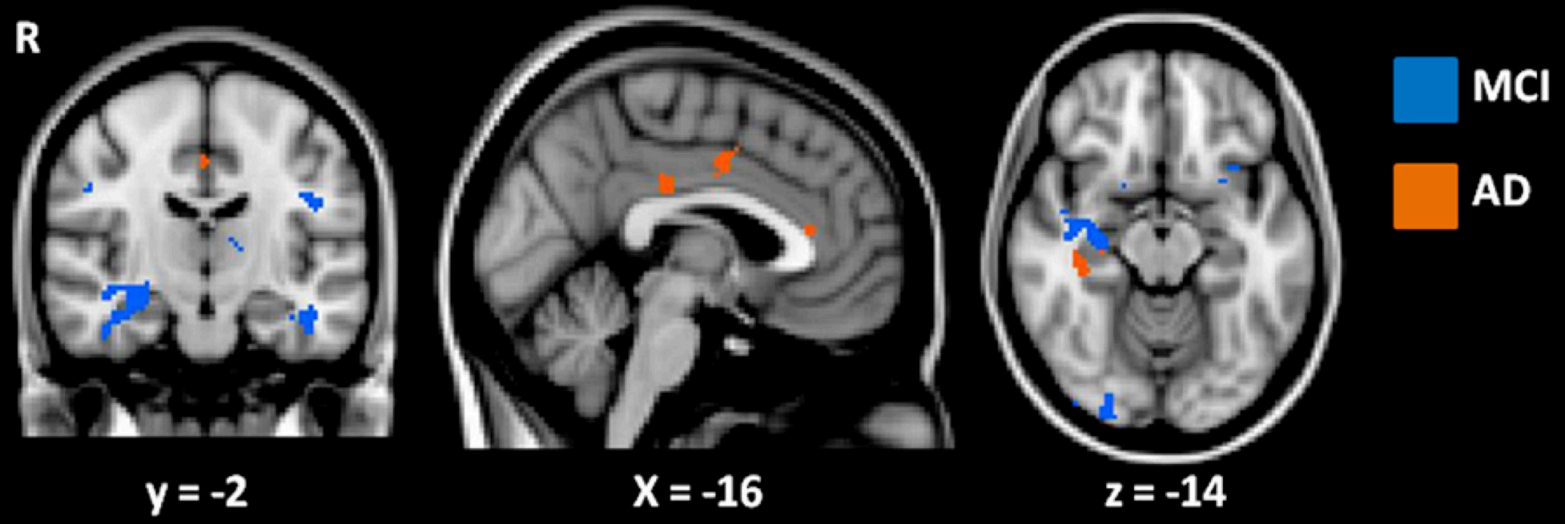
Results of VBM correlation analyses between AQ-D and gray matter density in MCI (in blue) and AD (in orange) groups. Results are displayed at *p* < 0.001 uncorrected. Images are shown in radiological convention. Coordinates are in MNI.

Further correlation analyses with AD-Q scores were performed separately for the AD and MCI subgroups to rule out an effect of disease severity. These analyses showed that the significant correlation between higher AQ-D scores and greater atrophy in structures of the medial temporal lobe persisted in both subgroups (*p* < 0.001, uncorrected, age and MMSE entered as covariates, Figure 4). In addition, in the AD group, higher AQ-D scores were correlated also with greater atrophy in the anterior and middle cingulate gyri, suggesting an involvement of these regions in anosognosia only when cognitive impairment is severe enough to reach the clinical diagnosis of dementia.

## Discussion

We investigated the neuropsychological and neuroanatomical correlates of anosognosia in patients with MCI and AD using a multidimensional approach. We found that the three most commonly used measurements of anosognosia, namely clinical rating (measured with CIRS), patient-informant discrepancy (measured with AQ-D), and performance discrepancy (measured with SAD scores on test of memory and executive function) are all independently associated with gray matter atrophy of medial temporal structures, particularly the right hippocampus.

We first investigated the behavioral correspondences of the three methods in order to examine their reciprocal relationships. Consistently with previous studies, we found high correlation between the two measures of anosognosia most frequently used in clinical settings that assess global, enduring and *offline* awareness, namely clinical rating and patient-informant discrepancy (Hannesdottir and Morris, 2007; Leicht et al., 2010; Clare et al., 2011). Among measures of discrepancy between performance on neuropsychological tests and patient's retrospective appraisal (indicated as performance discrepancy or self-appraisal discrepancy, SAD), which are usually considered *online* dynamic measures of anosognosia, only the one relative to the frontal assessment battery (SAD-FAB) correlated with patient-informant discrepancy (AQ-D). However, among all the tests used for calculating SAD scores, the FAB was the only one that included a battery of different sub-tests rather than a single test, therefore it is possible that in this specific case patients may have relied more on an enduring offline awareness system rather than on the immediate retrospective appraisal of actual performance when giving their judgments.

We then independently explored the specific association of each anosognosia measurement with focal gray matter atrophy and consistently found a significant involvement of the right hippocampus. More precisely, the more severe was the anosognosia score, the higher was the degree of atrophy in the right hippocampus, irrespectively of the method used to measure anosognosia. The association between hippocampus and measures of anosognosia was independent from patient's age or severity of cognitive decline, whose effect was accounted for in the imaging analyses, and remained significant also when considering MCI and AD patients separately.

The involvement of the hippocampus and other medial temporal lobe structures as key structures in anosognosia in patients with MCI and AD is consistent with several previous studies (for a recent review see Chavoix and Insausti, 2017). Specifically, the role of the right hippocampus also emerged in a post-mortem study correlating neuropathology and anosognosia in patients with AD: senile plaque density of the presubiculum of the right hippocampus was significantly higher in anosognosic relative to aware patients (Marshall et al., 2004). A large PET multicenter imaging study also showed that anosognosia was related to hypometabolism in right parahippocampal regions, suggesting a possible specific role of medial temporal lobe structures as “comparator” between current information and personal knowledge of cognitive abilities (Salmon et al., 2006).

But the relevance of the present study is that the three different methods most commonly used to assess anosognosia showed overlapping neuroanatomical correlates. This supports a unitary view on the mechanism leading to anosognosia in MCI and AD, which is in line with the Cognitive Awareness Model, a theoretical model of anosognosia first proposed by Agnew and Morris (Agnew and Morris, 1998; Morris and Hannesdottir, 2004), rather than supporting multicomponent/multilevel mechanisms of anosognosia. More precisely, the specific involvement of medial temporal structures is in line with the most recent revision of such theoretical model of anosognosia (Mograbi et al., 2009; Morris and Mograbi, 2013). This suggests that the pattern of memory impairment in MCI and AD may be responsible for the loss of components of autobiographical memory, which are the first steps necessary for forming a correct personal knowledge. This in turn is then necessary to update a more global, enduring, and on-line self-awareness system, whose neural correlates possibly involve the frontal cortex. In line with this, it is well known that the hippocampus and the medial temporal lobe have a role in supporting episodic and semantic components of autobiographical memory (Gilboa et al., 2005), that these structures are specifically involved in AD in its early phases (Tondelli et al., 2012), and that the hippocampus does not function as a unitary and isolated entity, but is part of several complex functional networks that variably adapt to disease (Zamboni et al., 2013b; Voets et al., 2014).

Interestingly, structures of the right medial temporal lobe were the only significantly correlates emerging when using measures of enduring, *offline* global awareness (i.e., clinical ratings and patient-informant discrepancy). Only when the correlates of patient-informant discrepancy (AQ-D) were studied in the AD patients only, significant correlations with atrophy also emerged in the anterior and mid-cingulate cortices. This result may be in line with previous studies using functional MRI that showed that patients with AD present loss of activation of medial prefrontal cortex in tasks requiring self-monitoring and self-awareness (Amanzio et al., 2011; Zamboni et al., 2013a; Perrotin et al., 2015).

When using measures of performance discrepancy (SAD scores), other regions of significant association between anosognosia and gray matter atrophy emerged in addition to the right hippocampus, which resulted from all the correlations. More precisely, performance discrepancy on verbal memory (SAD-RAVLT and SAD-Babcock Story Recall) was also associated with atrophy in the left hippocampus and amygdala. Performance discrepancy on visuo-spatial memory (SAD- Rey-Osterrieth Complex Figure-Recall) was also associated with atrophy in the precuneus and lateral occipital cortex. Performance discrepancy on executive and attentive functions (SAD-FAB and SAD-Stroop) was also associated with atrophy in the anterior cingulate, precentral gyrus and fronto-orbital cortex. This may suggest that the neural correlates of cognitive domain-specific anosognosia may include brain structures known to be directly involved in the specific cognitive function. Previous studies have hypothesized that patients may have domain-specific anosognosia for memory (Reed et al., 1993) and for a range of other possible symptoms including depression, apathy, and anxiety (Vasterling et al., 1995). Our results may suggest that the *on-line* dynamic awareness measured with the performance discrepancy method depends on the specific neural networks associated with the execution of the task itself. As postulated by the revision of the Cognitive Awareness Model (Morris and Mograbi, 2013), monitoring of performance may work at multiple levels, explaining the existence of domain-specific anosognosia: initially, at the first of these levels, sensory inputs providing information about performance are processed locally, in domain-specific modules, and only later are integrated centrally in the self-awareness system. Therefore, our findings from SAD scores may be again in line with this model and further support the idea that on-line monitoring relies on a widespread network in which local “spokes” specific for cognitive domain are interconnected with central “hubs” in the general awareness system.

However, it should be noted that, differently from several functional imaging studies that directly tested the neurocognitive mechanisms at the basis of self-awareness and anosognosia with experimental paradigms (Amanzio et al., 2011; Zamboni et al., 2013a; Perrotin et al., 2015), the present study simply tested associations between the most frequently used methods to measure anosognosia and brain structure, testing whether they capture the same variability in the degree of brain regional atrophy in our sample. Thus, our results capture rather basic and common features of anosognosia, and do deepen into the processes underpinning impaired awareness of symptoms in AD and MCI. Any mechanistic interpretation of our results should therefore be considered speculative.

One limitation of our study is that our sample of patients is relatively small and heterogeneous, since we included both AD and MCI patients. Future studies should be more selective in recruiting patients in order to distinguish potential specific features of anosognosia in different stages of AD progression, and possibly include subtler measures of disease severity as covariate of no interest. Another limitation is that, while we used validated questionnaires for clinical rating and patient-informant discrepancy, namely CIRS and AQ-D, there is no gold standard yet for measuring performance discrepancy (Ecklund-Johnson and Torres, 2005). As previously pointed out by other authors, the procedure most frequently used for performance discrepancy (namely, prediction of the number of remembered items from a list) is only suited to investigate self-appraisal discrepancy on memory performance (Leicht et al., 2010). We aimed instead to explore performance discrepancy in other cognitive domains beside verbal memory. Therefore, we followed the procedure suggested in previous studies (Leicht et al., 2010; Rosen et al., 2010), aware of the possible limitations such as the fact that SAD scores may potentially be influenced by motivation or unmeasured cognitive functions.

The strength of our study is that it combined behavioral and imaging investigations of anosognosia simultaneously measured in the same group of patients with the three most frequently used methods of assessments. The results were strikingly converging and highlighted the involvement of medial temporal lobe structures, in particular the right hippocampus, in the mechanism underlying anosognosia, however measured. These results have important theoretical implications in that contribute to an improved understanding of the neuroanatomical basis of anosognosia in MCI and AD. They also have direct practical implication as they suggest that anosognosia in MCI and AD can be reliably studied either through clinical questionnaires or through meta-cognitive methods.

## Author contributions

MT study design and concept, acquisition of imaging data, analysis and interpretation of data, draft and revision of the manuscript; AB study design, acquisition of neuropsychological data, data analysis and interpretation, revision of the manuscript; GV data analysis and interpretation, critical revision of the manuscript; CV acquisition of data, critical revision of the manuscript; AC study design and concept, acquisition of data, revision of the manuscript; PN study design and concept, study. supervision and funding, revision of the manuscript; GZ study design and concept, study funding, analysis and interpretation of data, draft and critical revision of the manuscript.

### Conflict of interest statement

The authors declare that the research was conducted in the absence of any commercial or financial relationships that could be construed as a potential conflict of interest. The reviewer LC and handling Editor declared their shared affiliation.
